# Carriage and within-host diversity of *mcr-1.1-*harbouring *Escherichia coli* from pregnant mothers: inter- and intra-mother transmission dynamics of *mcr-1.1*

**DOI:** 10.1080/22221751.2023.2278899

**Published:** 2023-12-17

**Authors:** Sharmi Naha, Priyanka Basak, Kirsty Sands, Rebecca Milton, Maria J. Carvalho, Shravani Mitra, Amrita Bhattacharjee, Anuradha Sinha, Suchandra Mukherjee, Bijan Saha, Pinaki Chattopadhyay, Partha Sarathi Chakravorty, Ranjan Kumar Nandy, Shanta Dutta, Timothy R. Walsh, Sulagna Basu

**Affiliations:** a Division of Bacteriology, ICMR-National Institute of Cholera and Enteric Diseases, Kolkata, India; b Institute of Infection and Immunity, Cardiff University, Cardiff, UK; c Department of Zoology, Ineos Oxford Institute of Antimicrobial Research, University of Oxford, Oxford, UK; d Centre for Trials Research, Cardiff University, Cardiff, UK; e Department of Medical Sciences, Institute of Biomedicine, University of Aveiro, Aveiro, Portugal; f Department of Neonatology, Institute of Post-Graduate and Medical Education & Research, Kolkata, India; g Department of Obstetrics & Gynecology, Institute of Post-Graduate and Medical Education & Research, Kolkata, India

**Keywords:** Colistin-resistant *Escherichia coli*, pregnant mother and neonatal gut carriage, *mcr-1.1*-bearing IncHI2, transmission dynamics of *mcr-1.1*, Illumina & MinION nanopore sequencing

## Abstract

Exchange of antimicrobial resistance genes via mobile genetic elements occur in the gut which can be transferred from mother to neonate during birth. This study is the first to analyse transmissible colistin resistance gene, *mcr*, in pregnant mothers and neonates. Samples were collected from pregnant mothers (rectal) and septicaemic neonates (rectal and blood) and analysed for the presence of *mcr*, its transmissibility, genome diversity, and exchange of *mcr* between isolates within an individual and across different individuals (not necessarily mother–baby pairs). *mcr-1.1* was detected in rectal samples of pregnant mothers (*n* = 10, 0.9%), but not in neonates. All *mcr*-positive mothers gave birth to healthy neonates from whom rectal specimen were not collected. Hence, the transmission of *mcr* between these mother-neonate pairs could not be studied. *mcr*-*1.1* was noted only in *Escherichia coli* (phylogroup A & B1), and carried few resistance and virulence genes. Isolates belonged to diverse sequence types (*n* = 11) with two novel STs (ST12452, ST12455). *mcr-1.1* was borne on conjugative IncHI2 bracketed between IS*Apl1* on Tn*6630*, and the plasmids exhibited similarities in sequences across the study isolates. Phylogenetic comparison showed that study isolates were related to *mcr*-positive isolates of animal origin from Southeast Asian countries. Spread of *mcr-1.1* within this study occurred either via similar *mcr*-positive clones or similar *mcr*-bearing plasmids in mothers. Though this study could not build evidence for mother–baby transmission but the presence of such genes in the maternal specimen may enhance the chances of transmission to neonates.

## Introduction

The human intestine accommodates a complex dynamic microbial community including antimicrobial-resistant Gram-negative bacteria harbouring resistance genes such as *bla*_TEM_, *bla*_CTX-M_, *qnrS*, etc. in both healthy and sick populations [1,2]. The intestine allows for the inter- and intra-species exchange of antimicrobial resistance genes (ARGs) primarily via different mobile genetic elements (MGEs) [3]. MGEs (plasmids, transposons, etc.) aid exchange of genes. Exchange of genes in the gut can occur due to antibiotic exposure or even without its use [3,4]. However, the use of antibiotics exerts selective pressure that facilitates the overgrowth of resistant bacteria.

Early in life, the newborn acquires microbes from the mother and the immediate environment [3,5,6]. Antibiotic consumption during pregnancy increases the risk of selection of antibiotic-resistant bacteria in the maternal microbiota which in turn may be passed on to the newborn during birth [2]. The acquisition of resistance genes from the mother or the hospital environment can have serious consequences for the newborn particularly those who are preterm or low birth weight [7]. Translocation of the bacteria, which includes those that are resistant, from the neonatal gut via the mysentric lymph node to the bloodstream can lead to sepsis [8].

Members of the Enterobacterales family such as *Escherichia coli*, commensals of the human gut, can serve as a common reservoir of different ARGs [3,9]. They have been found to exchange antibiotic-resistant plasmids and virulence genes among themselves [3]. In comparison to the Bacteroides or Firmicutes, *E. coli* are fewer in gut but have the ability to acquire and exchange genes with other commensal and transient bacteria [10]. Several resistance genes (*bla*_NDM_, *bla*_CTX-M_, *bla*_CMY_, *qnrS,* etc.) conferring resistance to different groups of antibiotics have been noted in *E. coli* [3,11].

In 2015, plasmid-mediated colistin resistance gene, *mcr-1*, emerged in China and *E. coli* served as the reservoir of this gene [12,13]. Other bacteria such as *Salmonella sp., Shigella sp., Klebsiella sp., Enterobacter cloacae*, etc. were also reported to harbour *mcr* genes [14]. *mcr-1* has been found in multiple plasmid types, *viz.* IncI2, IncHI2, IncX4, IncP, and IncF, signifying the efficient spread of this gene among various organisms and countries [15]. Apart from livestock, poultry, and aquaculture, the occurrence of *mcr* genes has been detected in humans [14] both in isolates causing infection and in colonizers, with a recent report of *mcr-1* gene in the gut of healthy human [15,16]. The presence of transmissible *mcr* gene in the healthy human gut, particularly in pregnant mother might expose neonates to bacteria harbouring the gene [3]. This would result in the emergence of resistance in neonates even in the absence of colistin exposure, ultimately leading to treatment failure.

Reports of *mcr* and their transmission dynamics in human gut is limited, more so in pregnant mothers and neonatal population. With the increased use of colistin as a last line resort and incidences of sepsis due to resistant bacteria, an understanding of colistin-resistant bacterial carriage and transmission in the gut is essential. Herein, we studied (i) carriage of *mcr* in the gut of mothers and sick newborns (not necessarily mother–baby pairs), and among neonatal blood isolates, (ii) inter- and intra-patient transmission dynamics of *mcr* and comparison of *mcr-*bearing organisms with susceptible isolates from the same sample, and (iii) phylogenetic relationships among the study isolates and with similar isolates across different Southeast Asian countries.

## Materials and methods

### Study design, collection of rectal swabs, and blood cultures at clinical site

In this study, samples collected during a multi-centric study named “Burden of Antibiotic Resistance in Neonates from Developing Societies (BARNARDS)” [2,17] have been used. BARNARDS study involved pregnant mothers and sick newborns who were admitted in IPGMER and SSKM Hospital, Kolkata. Rectal samples from all pregnant mothers but only sick neonates (no healthy neonates) were collected, so the mother–baby pairs were only present for sick neonates. Herein, we focused on analyzing carriage of the transmissible colistin resistance gene, *mcr*, and its transmission dynamics within the samples collected during the BARNARDS study.

**Figure 1.**
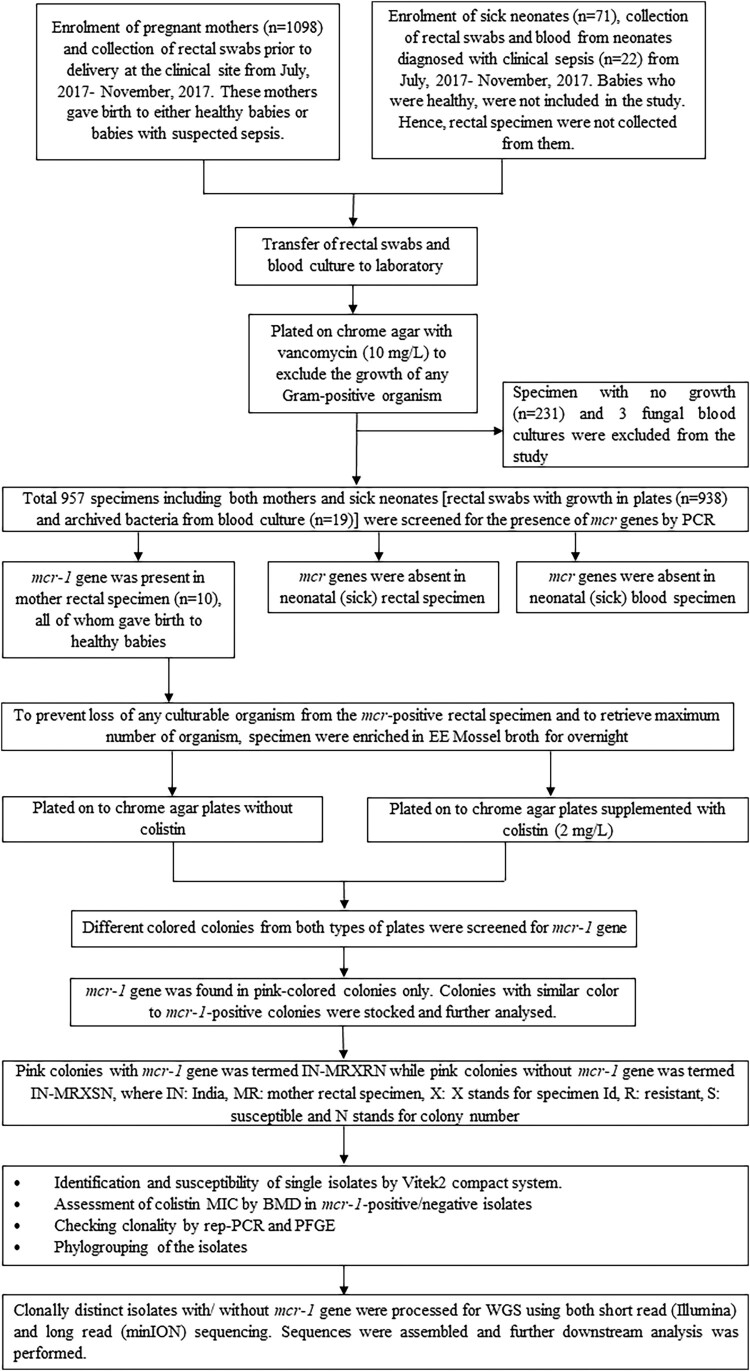
Schematic flow diagram of the study plan, summarizing sampling/laboratory/sequencing workflows.

During the study period (July to November 2017), women in labour or immediately postpartum were recruited, and rectal swabs were collected following consent. Neonates (inborn and outborn) when suspected with sepsis were enrolled, rectal swabs were collected and cultured isolates from blood were sent to laboratory. As a part of the BARNARDS study, socio-demographic information of the mothers and neonates were collected [17]. A schematic representation of the study design has been depicted in Figure 1.

### Processing of rectal swabs and blood cultures, and detection of transmissible colistin resistance gene, *mcr*

Processing of rectal swabs and blood culture have been described in Figure 1 and in supplementary methods. Briefly, rectal swabs were plated on vancomycin (10 mg/L) (MP Biomedicals, California, USA) supplemented chrome agar (CA) (BD BBL, MD, USA) and incubated (37°C, 18–24 h). Cultures from the primary inoculum of each plate and cultured isolates from neonatal blood were screened for *mcr* genes by polymerase chain reaction (PCR) as described previously [18]. Sample positive for *mcr* genes were further enriched in Enterobacteriaceae Enrichment (EE) Mossel broth (37°C, 18–24 h) (BD BBL) and plated on to CA supplemented with/without colistin sulphate (2 mg/L) (MP Biomedicals).

Colonies with different colours were picked from plates with/ without colistin, and again screened for *mcr* genes. Amplified PCR products were Sanger sequenced and stocked for further analysis [12]. Any *mcr*-negative colony of similar colour as the *mcr*-positive ones were collected from CA plates (without colistin) to check clonality with *mcr*-positive colonies.

### Identification and susceptibility of *mcr-*positive/*mcr-*negative isolates

Identification and susceptibility testing of all *mcr-*positive/*mcr-*negative isolates were done by Vitek2 compact system (BioMérieux, Marcy l'Etoile, France). For minimum inhibitory concentration (MIC) of colistin, broth microdilution (BMD) was carried out using colistin sulphate, following CLSI guidelines. Results were interpreted according to CLSI (2020) [19].

### Molecular typing of *mcr-*positive/*mcr-*negative isolates

More than one colony of same colour with *mcr* gene was stocked from each sample and were subjected to repetitive extragenic palindromic elements-PCR (rep-PCR) [20]. Additionally, all *mcr-*negative isolates (of same colour as the *mcr*-positive ones) were subjected to rep-PCR to check for similar clones. Clonality among *mcr-*positive isolates were determined by pulsed-field gel electrophoresis (PFGE) in a CHEF-DRIII apparatus (Bio-Rad Laboratories, Hercules, and CA) using XbaI macro digestion and visually interpreted as per Tenovar criteria [21]. With FP Quest software v4.5 (Biorad Laboratories Inc, Hercules, California, USA), a dendrogram was prepared using Dice coefficient and UPGMA (unweighted pair group method using arithmetic averages). Tolerance and optimization were set at 1.5% and isolates with ≥90% similarity were considered identical. The phylogenetic classification of the isolates was performed *via* phylogroup multiplex PCR as described previously [22].

### Transmissibility of plasmid-mediated *mcr*

Transfer of *mcr* was carried out in Az^r^ *E. coli* J53 (recipient) by solid-mating conjugation method. Transconjugants (TCs) were selected on Luria Bertani (LB) (BD BBL) agar plates supplemented with sodium azide (100 mg/L) (Sigma–Aldrich, St Louis, MO, USA) and colistin (2 mg/L). TCs retrieved were subjected to confirmation of *mcr* and other resistance genes by PCR, followed by assessment of colistin MIC by BMD.

Following this, plasmid typing was performed using PCR-based replicon typing (PBRT) [23] in both wild-type (WT) and TCs.

### Whole genome sequencing (WGS) of *mcr-*positive/*mcr-*negative isolates

Genomic DNA from *mcr*-positive/*mcr*-negative isolates was processed for paired-end WGS on an Illumina MiSeq. Selected isolates (based on genomic DNA quantity) were subject to additional long-read sequencing on a MinION (Oxford Nanopore Technology, UK) as described previously [24]. Further analysis of resistance, virulence, plasmid types, and sequence types was carried out by different online pipelines (Supplementary methods).

### Comparison of *mcr* bearing plasmids within the study isolates

Complete plasmid sequences of *mcr*-positive isolates were extracted using Bandage (v0.8.1), annotated using Prokka (v1.14.5), and mobile element finder (MGE) (v1.0.2). Sequences were aligned using Geneious (https://www.geneious.com/) and EasyFig (v2.2.5). Bacterial plasmid database (PLSDB) [25] was used to search for similar plasmid sequences and the plasmid with the greatest similarity (as determined by the match with the highest % nucleotide identity match to the *mcr* plasmid sequence from this study) was used as a reference sequence for sequence alignment analysis using GSAlign (v1.0.22).

### Comparative phylogenetic analysis of *mcr-*positive/*mcr-*negative isolates

A core genome alignment and maximum likelihood phylogenetic tree of isolates was performed using Roary (v3.12.0) and IQ-TREE (v2.0) with the GTR substitution model and 1000 bootstrap replicates. Phylogenetic trees were mid-rooted and annotated using iTOL(v5.7). Snippy (v4.6.0) was used to assess the number of single nucleotide polymorphisms (SNPs) between isolates of the same ST using an available hybrid genome as the local reference genome with –mincov 10 applied and trimmed paired end fastq used as input (–R1 –R2).

### Phylogenomic comparison of study isolates with *mcr-1* from Southeast Asia

A literature search was conducted during July–August 2021, WGS data of *mcr*-bearing *E. coli* from Southeast Asia was retrieved and fastq were assembled into genomes as described [24]. All genomes were screened for the presence of *mcr-1*. ClermonTyping was used to determine *in silico* phylogroups [26,27], Prokka (v1.14.5) was used to annotate the assemblies and Panaroo (v1.2.8; –clean-mode [moderate], –core parameters applied) was used to create the core genome alignment. IQ-TREE (v2.0) was used to generate the maximum likelihood phylogenetic tree with parameters as described previously [24].

## Results

### Identification of bacterial isolates with *mcr*

*mcr-1* was found in rectal samples of ten pregnant mothers (*n* = 10/1169), indicating a low prevalence (0.9%). These 10 mothers had healthy babies and hence the paired neonatal sample was not collected. None of the other sick neonates from whom rectal samples were collected harboured *mcr-1*. Sanger sequencing of *mcr-1* revealed it to be *mcr-1.1* and was found only in *Escherichia coli*. *E. coli* lacking *mcr-1.1* from those samples that harboured *mcr-*positive isolates were also analysed. Fourteen *mcr-1.1*-positive *E. coli* and 25 *mcr*-negative *E. coli* were isolated from 10 mothers (Table 1). Few mothers had more than one isolate carrying *mcr-1.1*. Other colonies found in the sample were *Klebsiella pneumoniae*, *Pseudomonas aeruginosa*, and *Acinetobacter baumannii*, but none carried *mcr-1*.

**Table 1.** Colistin susceptibility and genotypic characterization of *mcr-1­.1-*positive *Escherichia coli*, their transconjugants (TCs), and other susceptible *Escherichia coli* from the same rectal specimen.

Strain Id		Genomic characterization									Other bacterial species detected in the same rectal specimen
	Colony Id	Phylo group	Sequence type (ST)	AMR phenotype	Colistin MIC (mg/ L)	*mcr* genes	Resistance determinants present/ transferred	Plasmid types	Plasmid ST (pST) of *mcr-1.1*-bearing plasmid	Virulence genes	
IN-MR-361	S1	A	181	–	0.125	Absent	*mdf(A)*	IncFIA (HI1), IncFIB (K)	NA	*gad, terC*	No
	R1	A	181	AMP, SXT	16	*mcr-1.1*	*bla*_TEM-1_, *bla*_EC-15_*, aadA1, aadA2, aph(3'')-Ib, aph(3')-Ia, aph(6)-Id, cmlA1, dfrA14, floR, mef(B), mph(A), sul3, tet(34), tet(A)*	IncFIA (HI1), IncFIB (K), IncHI2, IncHI2A	NA	*gad, terC*	
	R1.TC	ND	ND	AMP	16	*mcr-1.1*	*bla*_TEM-1_	IncHI2 (∼240 kb)	pST4	ND	
IN-MR-362	S1	A	542	AMP, NAL, CIP	0.125	Absent	*bla*_TEM-1C_, *mdf(A), tet(A)*	Absent	NA	Absent	No
	S2	A	**12454**	AMP, SXT	0.0625	Absent	*bla*_TEM-1B_, *qnrS13, mdf(A), aph(6)-Id, mph(A), dfrA14, sul3, tet(A)*	IncX1	NA	*gad, ompT, sitA, terC*	
	R1	A	**12452**	AMP, CXM, CRO, FEP, NAL, CIP, SXT	16	*mcr-1.1*	*bla*_CTX-M-15_, *bla*_TEM-1_, *bla*_EC-15_, *qnrS1, aadA1, aadA2, aph(3'')-Ib, aph(3')-Ia, aph(6)-Id, cmlA1, dfrA14, floR, mph(A), sul3, tet(34), tet(A)*	IncHI2, IncHI2A	NA	*gad, iss, kpsE, kpsMII, terC*	
	R1.TC1	ND	ND	AMP	8	*mcr-1.1*	*bla*_TEM-1_	IncHI2 (∼240 kb)	NF	ND	
	R1.TC2	ND	ND	AMP	8	*mcr-1.1*	*bla*_TEM-1,_*qnrS1*	IncHI2 (∼240 kb)	NF	ND	
IN-MR-364	S1	A	**12454**	AMP, SXT	0.25	Absent	*bla*_TEM-1B_, *qnrS13, mdf(A), aph(6)-Id, tet(A), mph(A), sul3, dfrA14*	IncX1	NA	*gad, ompT, sitA, terC*	*Klebsiella pneumoniae*
	S2	A	**12453**	AMP, CXM	0.25	Absent	*bla*_CTX-M-15_, *qnrS1, mdf(A)*	Absent	NA	Absent	
	S3	A	2491	CXM, CFP/SUL	≤0.5	Absent	*bla*_CTX-M-15_, *bla*_TEM-1B_, *qnrS13, mdf(A), aph(6)-Id, aadA1b, tet(A), sul3, sul1, mph(A), qacE*	Col440I, IncFIB (K), IncX1	NA	*capU, gad, ompT, sitA, terC*	
	R1	A	674	AMP, CXM, CRO, SXT	8	*mcr-1.1*	*bla*_CTX-M-15_, *bla*_EC-15_, *qnrS1, aadA1, aadA2 bla*_TEM-1_, *aph(3'')-Ib, aph(3')-Ia, aph(6)-Id, cmlA1, floR, mph(A), sul3, tet(34), tet(A)*	IncHI2, IncHI2A	NA	*capU, gad, terC*	
	R1.TC	ND	ND	AMP	8	*mcr-1.1*	*bla*_TEM-1_	IncHI2 (∼240 kb)	Unknown	ND	
IN-MR-569	S1	B1	12387	SXT	0.25	Absent	*qnrS1, mdf(A), tet(A)*	IncFIB	NA	*etpD, gad, iss, lpfA, ompT, terC, traT*	*Klebsiella pneumoniae*
	S2	B1	366	–	0.25	Absent	*qnrS1, mdf(A), tet(A)*	IncFIB, IncFII	NA	*etpD, gad, iss, lpfA,* ompT, *terC, traT*	
	S3	A	2705	AMP, CXM, CRO	0.25	Absent	*qnrS1, mdf(A), tet(A), dfrA14, sul2*	IncX2, p0111	NA	*gad, ompT, terC*	
	R1	A	**12455**	SXT	8	*mcr-1.1*	*bla*_EC-15*,*_*qnrS1, dfrA14, tet(34), tet(A)*	IncFIA (HI1), IncFIB (K), IncX2, IncX4, p0111	NA	Absent	
	R1.TC	ND	ND	–	4	*mcr-1.1*	–	IncX4 (33 kb)	Unknown	ND	
IN-MR- 674	S1	B1	196	–	0.0625	Absent	*mdf(A)*	Absent	NA	*capU, gad, lpfA, terC*	*Pseudomonas aeruginosa, Acinetobacter baumannii*
	S2	B1	3640	–	0.0312	Absent	*mdf(A)*	Absent	NA	*gad, lpfA, sitA, terC*	
	R1	A	2705	AMP, SXT	8	*mcr-1.1*	*bla*_TEM-1_, *bla*_EC_, *qnrS1, aadA1, aadA2, aph(3'')-Ib, aph(3')-Ia, aph(6)-Id, cmlA1, dfrA14, floR, mef(B), mph(A), sul2, sul3, tet(34), tet(A)*	IncHI2, IncHI2A, IncN, IncX1, p0111	NA	*gad, neuC, ompT, terC*	
	R1.TC	ND	ND	AMP	8	*mcr-1.1*	*bla*_TEM-1_	IncHI2 (∼241 kb)	pST4	ND	
IN-MR-680	S1	B1	4038	–	0.0312	Absent	*mdf(A)*	Absent	NA	*gad, lpfA, terC*	*Klebsiella pneumoniae*
	S2	A	4995	–	0.0156	Absent	*mdf(A)*	Col, IncFIB (K), IncFII	NA	*gad, lpfA, terC*	
	S3	A	48	AMP	0.25	Absent	*bla*_TEM-1B_, *qnrS4, mdf(A), aph(3'')-Ib, aph(6)-Id, sul2, tet(A)*	Col, p0111	NA	*gad, terC*	
	R1	A	2705	AMP, SXT	8	*mcr-1.1*	*bla*_TEM-1_, *bla*_EC_, *qnrS1, aadA1, aadA2, aph(3'')-Ib, aph(3')-Ia, aph(6)-Id, cmlA1, dfrA14, floR, mef(B), mph(A), sul2, sul3, tet(34), tet(A)*	IncHI2, IncHI2A, IncN, IncX1, p0111	NA	*gad, neuC, ompT, terC*	
	R1.TC	ND	ND	–	8	*mcr-1.1*	–	IncHI2 (∼241 kb)	pST4	ND	
IN-MR-683	S1	B1	48	AMP	0.0312	Absent	*qnrS1, mdf(A), aadA5, dfrA17, tet(A)*	IncY	NA	*gad, lpfA, terC*	*Klebsiella pneumoniae*
	S2	A	**12457**	AMP	0.0156	Absent	*bla*_TEM-1B_, *qnrS4, mdf(A), aph(6)-Id, aph(3'')-Ib, sul2, tet(A)*	p0111	NA	*gad, terC*	
	S3	G	174	–	0.25	Absent	*mdf(A)*	Absent	NA	*gad, chuA, iss, lpfA, ompT, terC, usp*	
	S4	B1	3594	–	0.25	Absent	*mdf(A)*	IncI1-I (Alpha)	NA	*gad, lpfA, terC*	
	R1	A	2705	AMP, SXT	8	*mcr-1.1*	*bla*_TEM-,_*bla*_EC_, *qnrS1, aadA1, aadA2, aph(3'')-Ib, aph(3')-Ia, aph(6)-Id, cmlA1, dfrA14, floR, mef(B), mph(A), sul2, sul3, tet(34), tet(A)*	IncHI2, IncHI2A, IncN, IncX1, p0111	NA	*gad, neuC, ompT, terC,*	
	R1.TC	ND	ND	–	8	*mcr-1.1*	–	IncHI2 (∼241 kb)	pST4	ND	
IN-MR-725	S1	C	652	–	0.125	Absent	*mdf(A)*	IncY	NA	*gad, lpfA, terC,capU,*	*Klebsiella pneumoniae*
	S2	A	6856	AMP, CIP, NAL, SXT	0.25	Absent	*qnrS13, mdf(A), dfrA14, tet(A)*	p0111	NA	*gad, terC*	
	R1	B1	2178	AMP, NAL	8	*mcr-1.1*	*bla*_TEM-1_, *bla*_EC-18_, *aadA1, aadA2, cmlA1, sul3, tet(34)*	IncFIA, IncFIB (pB171), IncHI2, IncHI2A	NA	*gad, lpfA, terC, traT*	
	R1.TC	ND	ND	–	8	*mcr-1.1*	–	IncHI2 (∼216 kb)	pST4	ND	
	R2	B1	9421	AMP	8	*mcr-1.1*	*bla*_TEM-1_, *bla*_EC-18_, *aadA1, aadA2, cmlA1, sul3, tet(34)*	IncFIB (K), IncFII, IncHI2, IncHI2A, IncY	NA	*gad,lpfA,terC*	
	R2.TC	ND	ND	–	8	*mcr-1.1*	–	IncHI2 (∼216 kb)	pST4	ND	
	R3	B1	101	AMP, SXT	8	*mcr-1.1*	*bla*_TEM-1,_*bla*_EC-18_, *qnrS1, aadA1, aadA2, cmlA1, dfrA15, sul1, sul3, tet(34), tet(A)*	IncFIB (AP001918), IncFIC (FII), IncHI2, IncHI2A, IncX1	NA	*gad, cba, cia, cib, cma, cvaC, hlyF, iroN, iss, iucC, iutA, lpfA, ompT, sitA, terC, traT, tsh*	
	R3.TC	ND	ND	–	8	*mcr-1.1*	*qnrS1*	IncHI2 (∼216 kb)	pST4	ND	
IN-MR-727	S1	D	394	–	0.125	Absent	*mdf(A)*	Absent	NA	*air, chuA,eilA, gad, iss, kpsE, kpsMII_K5, lpfA, ompT, terC*	No
	S2	B1	3998	AMP	0.25	Absent	*bla*_TEM-1B_, *qnrB7*, *mdf(A), tet(A), sul2*	IncFIA (HI1), IncHI1A, IncHI1B (R27)	NA	*gad, lpfA, terC*	
	S3	B1	641	–	0.25	Absent	*mdf(A)*	IncFIB (K), IncFII	NA	*gad, lpfA, terC*	
	R1	A	1286	AMP, SXT	8	*mcr-1.1*	*bla*_TEM-1_, *bla*_EC-15,_*qnrS1, aadA1, aadA2, cmlA1, dfrA14, sul2, sul3, tet(34), tet(A)*	IncHI2, IncHI2A, IncY	NA	*gad, iss, terC*	
	R1.TC	ND	ND	–	8	*mcr-1.1*	*qnrS1*	IncHI2 (∼216 kb)	pST4	ND	
IN-MR-750	S1	D	394	–	0.5	Absent	*mdf(A)*	Absent	NA	*air, chuA, eilA, gad, iss, kpsE, kpsMII_K5, lpfA, ompT, terC*	*Acinetobacter baumannii*
	S2	B1	1125	–	0.5	Absent	*mdf(A)*	Absent	NA	*air, chuA, eilA, gad, lpfA, terC*	
	R1	D	394	–	16	*mcr-1.1*	*bla*_EC-8_, *dfrA14, mph(A), tet(A)*	IncHI2, IncHI2A	NA	*air, chuA, eilA, gad, iss, kpsE, kpsMII_K5, lpfA, ompT, terC*	
	R1.TC	ND	ND	–	16	*mcr-1.1*	–	IncHI2 (∼217 kb)	pST4	ND	
	R2	B1	515	AMP	16	*mcr-1.1*	*bla*_TEM-1_, *bla*_EC-15_, *aadA1, aadA2, cmlA1, sul3, tet(34)*	IncHI2, IncHI2A	NA	*gad, terC*	
	R2.TC	ND	ND	AMP	8	*mcr-1.1*	*bla*_TEM-1_	IncHI2 (∼216 kb)	pST4	ND	
	R4	D	394	AMP	8	*mcr-1.1*	*bla*_TEM-1_, *bla*_EC-8_, *aadA1, aadA2, cmlA1, sul3*	IncHI2, IncHI2A	NA	*air, chuA, eilA, gad, iss, kpsE, kpsMII_K5, lpfA, ompT, terC*	
	R4.TC	ND	ND	AMP	8	*mcr-1.1*	–	IncHI2 (∼216 kb)	pST4	ND	

Abbreviations: IN-MR (IN-India, MR-maternal rectal specimen), resistant (R), susceptible (S), minimum inhibitory concentration (MIC), Ampicillin (AMP), Cefuroxime (axetil or sodium) (CXM), Ceftriaxone (CRO), Cefoperazone (CFP)/Sulbactam (SUL), Cefepime (FEP), Nalidixicacid (NAL), Ciprofloxacin (CIP), Trimethoprim-sulfamethoxazole (SXT), Transconjugants (TC), kilobase pairs (kb), not applicable (NA), not done (ND), not found (NF). Shaded rows indicate *mcr-1.1* bearing wild-type strains. Bold-faced STs are novel to this study.

### Socio-demographic details of mothers possessing *mcr-1.1*-positive *E. coli*

Mothers who harboured *mcr-1.1* gave birth to term, singleton, healthy babies, with no birth complications. Since, the babies were healthy, rectal swabs were not collected from these neonates and hence, not assessed for the presence of *mcr-1.1*. No mother received antibiotic treatment, nor visited traditional, or private healthcare or were hospitalized or travelled outside the local province prior to enrolment. Most mothers (*n* = 7) were from urban areas while three mothers were from rural areas.

### Antibiotic susceptibility of *E. coli* isolates collected from *mcr-1.1*-positive maternal sample

Both *mcr-*positive and *mcr-*negative isolates were susceptible to most of the antibiotics tested but were resistant to ampicillin and trimethoprim-sulfamethoxazole (Table 1). Few were additionally resistant to cefuroxime, ceftriaxone, cefoperazone/sulbactam, cefepime, nalidixic acid, and ciprofloxacin. However, *mcr-*positive isolates were also resistant to colistin (MIC: 8–16 mg/L), while *mcr-*negative isolates were susceptible (0.015–2 mg/L) (Table 1).

### Molecular typing of *E. coli* from *mcr-1.1*-positive maternal sample

rep-PCR showed 11 distinct band patterns in 14 *mcr*-positive isolates, while band patterns were all diverse in case of 25 *mcr*-negative isolates. All the distinct isolates were proceeded with WGS.

**Figure 2.**
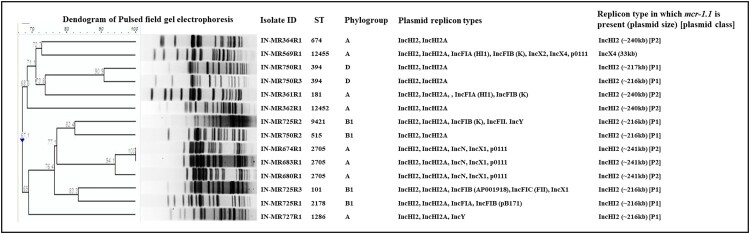
Molecular typing and plasmid replicons of *mcr-1.1* bearing *Escherichia coli*. Abbreviation: Indian maternal rectal sample (IN-MR), resistant (R), sequence type (ST), incompatibility (Inc), kilobase (kb), plasmid 1 (P1), plasmid 2 (P2).

PFGE revealed that *mcr-*positive isolates were clonally distinct with few indistinguishable isolates such as IN-MR674R1, IN-MR680R1 & IN-MR683R1; and IN-MR750R1 and IN-MR750R4 (Figure 2).

*mcr-*positive isolates belonged to diverse STs (11 STs among 14 isolates), of which ST12452 and ST12455 were novel (Table 1). *mcr-*negative isolates were also diverse (22 STs), with three novel STs (ST12453, ST12454, and ST12457) (Table 1). STs such as ST181, ST394, and ST2705 were common in both types of *E. coli*.

### Comparison of resistance, virulence, plasmid profiles, and phylogroups among both types of *E. coli* based on WGS

*mcr-*positive isolates harboured different β-lactamase resistance genes; fluoroquinolone, aminoglycoside, sulphonamide, macrolide, chloramphenicol, tetracycline, and trimethoprim resistance genes (Table 1). Most of the *mcr-*negative isolates (*n* = 17, 68%) did not bear β-lactamase genes but had a multidrug resistance gene, *mdf(A).* Few had β-lactamase genes (*bla*_TEM-1_, *bla*_CTX-M-15_) (*n* = 8), along with fluoroquinolone resistance genes (Table 1). None of the study isolates harboured carbapenemases.

Carriage of virulence genes within *mcr*-positive and *mcr*-negative isolates was nearly same (Table 1). *gad* (glutamate decarboxylase) was prevalent among both groups of isolates, followed by *terC* (tellurite resistance gene). Presence of different serum and complement resistance genes were noted in many *mcr-*positive and few *mcr-*negative isolates (Table 1). However, IN-MR569R1 and few *mcr-*negative didn’t show the presence of any virulence genes at all. Most of the study isolates belonged to phylogroup A (*mcr-*positive = 8 and *mcr-*negative = 11) and B1 (*mcr-*positive = 4 and *mcr-*negative = 10) (Table 1).

Both *mcr-*positive and *mcr-*negative isolates possessed different plasmid replicons such as IncFIA, IncFIB, IncFII, IncX1, IncX2, IncX4, p0111, IncN, IncY, IncHI1A, IncHI1B, IncI1-I (alpha), Col, and Col440I (Table 1). *mcr-*positive isolates additionally harboured IncHI2, IncHI2A, and IncX4.

### Transmissibility and characterization of *mcr-1.1* plasmid

Conjugal transfer of *mcr-1.1* was successful with co-transfer of *bla*_TEM,_ and *qnrS* in various combinations among transconjugants (TCs). Colistin MIC of TCs and wild-type isolates were found to be similar (Table 1).

**Figure 3.**
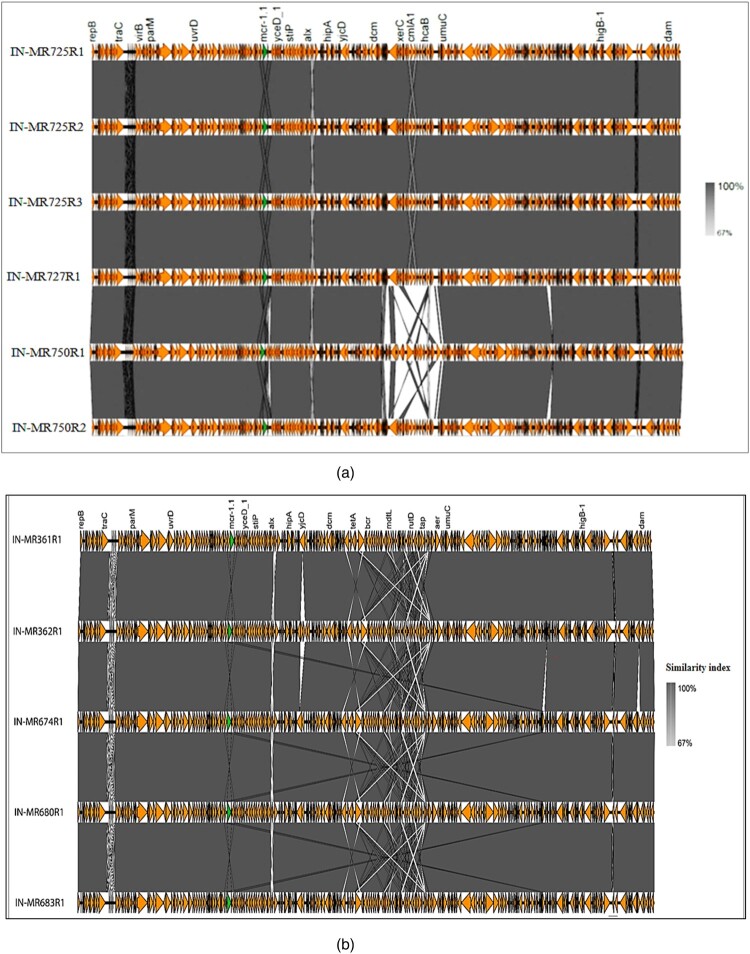
Alignment of *mcr-1.1-*bearing IncHI2 plasmid sequences of the study isolates. (a) ∼216 kb: IN-MR750R1 and IN-MR750R4 were identical, hence in this figure only IN-MR750R1 has been included. (b) ∼240 kb: Due to poor assembly issue, IN-MR364R1 was excluded from this analysis. Yellow ochre-coloured arrows: different genes, green arrow: *mcr-1.1*. Shaded regions: percentage similarities.

*mcr-1.1* was carried on IncHI2 plasmid (∼216–240 kb), except for one (IN-MR569R1) which harboured *mcr-1.1* in IncX4 (∼33 kb) (Table 1). Plasmid types found in TCs as evaluated by PBRT corroborated with WGS data. All IncHI2 plasmids belonged to pST4 (Table 1). Out of 14 *mcr-*positive isolates, 13 complete plasmids including the IncX4 plasmid were assembled from long-read sequencing. Two groups of IncHI2 plasmids were noted, ∼216 kb (plasmid 1, P1) found in multiple isolates collected from three samples and the other ∼240 kb (plasmid 2, P2) found in six different samples (Table 1, Figure 3a,b). All P1s (*n* = 7) when aligned against pCFSAN061771 (Egypt, accession: CP042898.1), were found to be identical to each other (Figure 3a) with an average nucleotide identity (ANI) of 93.07% to pCFSAN061771 (455 single nucleotide variants, SNVs; 8 insertions, and 5 deletions). P1 from IN-MR750R1 had an ANI >99.88% to pCFSAN061771 and was slightly larger (∼217 kb) (Figure 3a), showing some variations. Similarly, all P2s (*n* = 5) when aligned against a plasmid of an *E. coli* from Saudi Arabia (NZ_CP022735.1) exhibited >99.9% ANI. P2s from different mothers were within 50 pairwise SNVs (Figure 3b).

**Figure 4.**
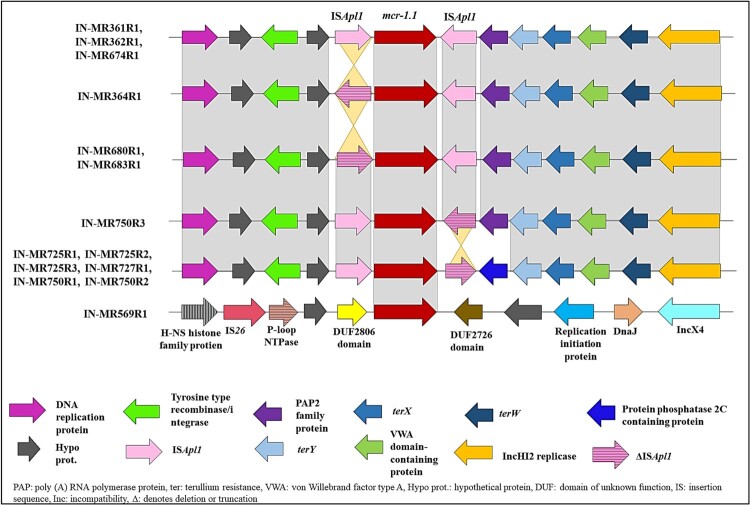
Genetic environment of *mcr-1.1* found among study isolates. Genes and their corresponding transcription orientations are indicated by horizontal arrows. Grey shaded region: homology, light yellow shaded region: inversion.

*mcr-1.1* in P1 and P2 was bracketed between IS*AplI* upstream and downstream within a composite transposon, Tn*6330*, with variations in orientation and truncation of IS*Apl1* in few isolates (Figure 4). Genetic environment of *mcr-1.1* of study isolates when compared with global *mcr-1*, revealed a variation in the arrangement of the genes, that is, IS*Apl1*-*mcr-1.1*-IS*Apl1*-*pap2* instead of IS*Apl1*-*mcr-1.1*-*pap2*-IS*Apl1* (Figure 4).

*mcr-1.1* when present in IncX4 plasmid (∼33 kb) did not have IS*AplI* or other IS elements in the vicinity, rather IS*26* was noted in the same plasmid (Figure 4). IncX4 plasmid when aligned with other previously described plasmid sequences, produced multiple identical hits on PLSDB with *mcr-1* isolates from China, Laos, and Vietnam.

### Transmission of *mcr-1.1* within gut microbiome

**Figure 5.**
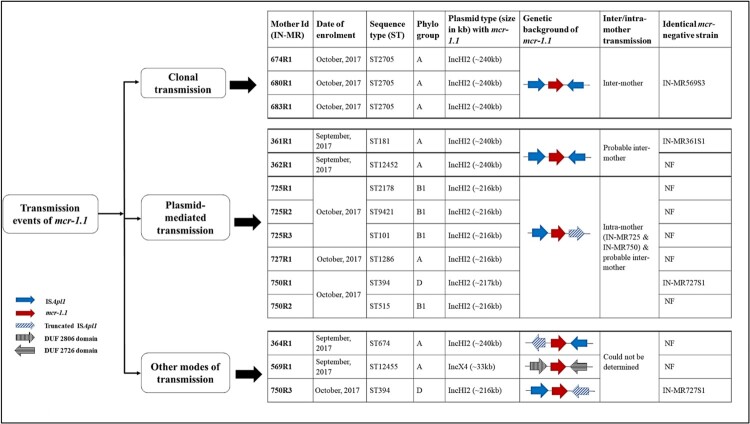
Transmission events of *mcr-1.1* gene occurring in different maternal samples. Abbreviation: India-Maternal rectal specimen (IN-MR), resistant (R), susceptible (S), not found (NF), incompatibility (Inc).

Within the study population, intra- and inter-gut transmission of *mcr-1.1* was studied. We compared isolates in terms of their typing patterns (PFGE), STs, SNPs, and *mcr-1.1*-bearing plasmids in individual or different maternal samples. We analysed transmission of *mcr-1.1* from two aspects: (i) clonal spread, where *mcr-*positive isolates of same STs were isolated from different mothers, and (ii) plasmid-mediated spread, where *mcr*-carrying similar plasmids belonging to different STs were isolated from individual or different mothers (Figure 5). When assessing the clonal spread, *mcr-*negative isolates belonging to similar STs of *mcr-*positive isolates were also compared.

**Figure 6.**
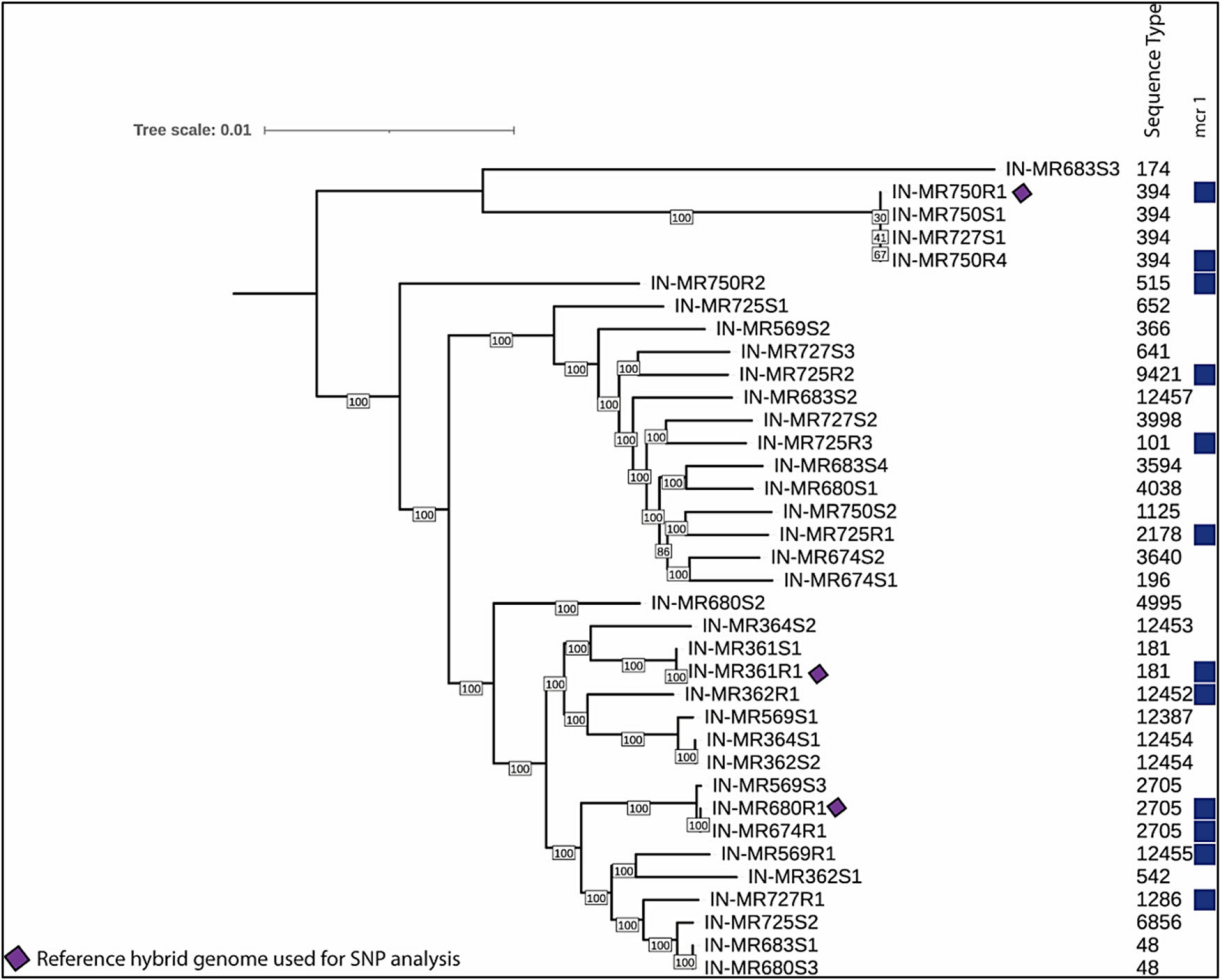
A core genome phylogenetic tree summarizing the isolates with short-read WGS available from this study. Due to contamination and poor assembly issue, IN-MR364R1 and IN-MR683R1 were excluded from this analysis.

Isolates IN-MR674R1, IN-MR680R1, IN-MR683R1 collected from three mothers admitted within a time frame of 0–1 days (Figure 5) belonged to ST2705, phylogroup A, and shared >90% similarity (PFGE) (Table 1, Figure 2). These isolates clustered together in core genome phylogenetic tree and IN-MR674R1, IN-MR680R1 were 22 SNPs distant from each other (Figure 6). These results indicated a possible inter-mother transfer of clonal isolates with *mcr-1.1*.

To understand the transmission of *mcr-1.1* through plasmids, we compared *mcr-1.1*-bearing IncHI2 plasmids in individual/different mothers. Sequences of *mcr-1.1*-bearing IncHI2 plasmid within five mothers (IN-MR361, IN-MR362, IN-MR725, IN-MR727, and IN-MR750) showed significant similarities. IN-MR361 (ST181) and IN-MR362 (ST12452) despite belonging to different STs, carried a similar *mcr-1.1* plasmid (240 kb) with identical genetic environment (Table 1, Figures 3b, 4, 5). Likewise, *mcr-1.1*-bearing IncHI2 plasmid of *E. coli* (ST1286) isolated from IN-MR727 also showed similarity with plasmids of *E. coli* from IN-MR725 and IN-MR750 (Table 1, Figures 3a, 4, and 5). These mothers were distantly related to each other as per core genome SNP phylogeny (Figure 6) but harboured a similar plasmid (Figure 5). Both horizontal transmission of *mcr-1* through other *E. coli*/ bacteria among different mothers or independent acquisition of similar plasmids at different points of time beyond the hospital environment are possible. However, this cannot be definitively assessed by the findings of this study. In some mothers, more than one diverse *E. coli* isolate belonging to different STs were found – IN-MR725 (ST101, ST2178, ST9421) and IN-MR750 (ST394, ST515), exhibiting similar *mcr-1.1*-bearing IncHI2 plasmids (∼216 kb) with same genetic environment (Table 1, Figures 3a, 4, 5). This indicates a possible transmission of plasmid within an individual host (intra-mother transmission).

Three other independent acquisitions of *mcr-1.1*-bearing plasmid/ bacteria were noted, one with *mcr-1.1* in IncX4 plasmid, and the other two with *mcr-1.1* in IncHI2 having a genetic environment distinct from others that are discussed above (Figure 5).

Few *mcr-*negative isolates identical to *mcr-*positive isolates in terms of STs and core genome were also isolated but they lacked IncHI2 and IncX4 (Table 1, Figures 5 and 6).

### Phylogenomic comparison of *mcr-1-E. coli* in Southeast Asia

**Figure 7.**
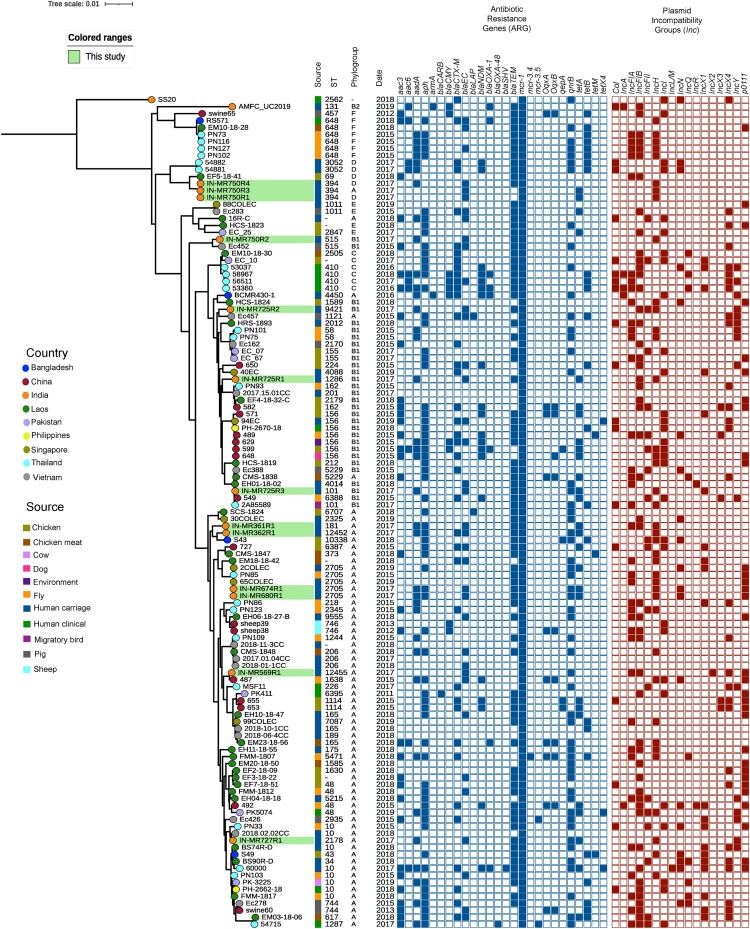
Core genome phylogenetic tree of *mcr-1-E. coli* collected from published studies in Southeast Asia including those from the present study. Sequence type (ST), green shading: study isolates. IN-MR364R1 and IN-MR683R1 were excluded due to contamination and poor assembly.

Core genome phylogeny was built involving genomic data from Southeast Asian *mcr-1*-positive *E. coli* (*n* = 106) collected between 2011 and 2019. This included isolates from 20 studies along with 12 *mcr-*positive isolates of this study (Figure 7). The isolates belonged to diverse sources including blowflies (*n* = 20), chicken (meat and stool, *n* = 30), dairy cattle farm (*n* = 1), dogs (*n* = 1), environment (*n* = 1), human carriage (*n* = 29), human clinical samples (*n* = 12), migratory birds (*n* = 1), pigs (*n* = 9), and sheep (*n* = 2) (Figure 7). Isolates were distributed in over 70 STs, with ST10, ST48, ST156, ST410, and ST648 being the most frequent. *mcr-*positive isolates of this study were found dispersed throughout the phylogeny indicating a wide diversity of *mcr-1 E. coli* isolates across the species.

Southeast Asian *mcr-1* collection harboured *bla*_CTX-M_ (*n* = 40), *bla*_NDM_ variant (*n* = 20), *bla*_OXA-48_ (*n* = 1), *tet(X4)* (*n* = 3), *mcr-3* (*n* = 3) in addition to the *mcr-1* gene. Whilst none of the study isolates within this study contained carbapenemase or *tet(X4)*, although few possessed other ARGs. Though different plasmid replicons were seen in the analysed genomes, *mcr-1* was principally present in IncHI2 or IncX4. Isolates possessed either IncHI2 or IncX4 but never both. Some isolates from Vietnam did not show the presence of any plasmid, indicating the occurrence of chromosomal *mcr-1* gene.

Analysed isolates belonged to phylogroup A (*n* = 57, 54%) followed by phylogroup B1 (*n* = 30, 28%), F (*n* = 7), C (*n* = 6), D (*n* = 5), and one case of B2. Likewise, study isolates also belonged to phylogroup A and B1.

Study strains showed closeness with strains from various countries of Southeast Asia *viz*. Bangladesh, China, Thailand, Singapore, Vietnam, and Laos (Figure 7). Most of the study isolates showed resemblances with *E. coli* isolated from chicken (*n* = 5) or human carriage (*n* = 4) and few from pig and blow flies. This shows that *mcr*-positive isolates circulate among different origins (mainly food animals), emphasizing its presence within the food chain.

## Discussion

The passage of antibiotic-resistant organisms from mother to the neonate is of concern. The pristine and immature gut of the neonate, particularly for those who are premature and low-birth weight, can allow the translocation of resistant organisms to the bloodstream leading to sepsis [28]. *mcr-1.1* was isolated from maternal rectal samples only, with very low prevalence (0.9%). No *mcr* was noted in gut samples or in cultured blood from neonates suspected with sepsis. Mothers with *mcr-1.1* had healthy babies and since samples were not collected from healthy neonates, the presence of *mcr* in the healthy neonatal gut could not be assessed. Hence, the possibility of mother-to-baby transmission of *mcr*-harbouring *E. coli* could not be studied.

Carriage of *mcr* in this study is much lower than that reported from China (2.08–6.2%), Switzerland, and France, but higher than Netherlands (0.35%) [29]. Colistin resistance is low in India and a study on neonatal blood isolates over a period of 12-years from the same unit also exhibited low prevalence of colistin resistance (2.8%) with no *mcr* genes [30]. Such low resistance may be attributed to the limited use of colistin in clinical settings. Absence of selection pressure (colistin) probably restricted the spread of *mcr-1* in clinical settings as studies from India documented fewer reports of *mcr-1* from human clinical isolates compared to food items such as meat, poultry, and environmental isolates [31,32]. Since 2019, restrictions on colistin usage in India within animal industries have attributed to low carriage of *mcr-1* [32].

In India, *mcr-1* was reported first in *Klebsiella pneumoniae* [33] and later in *E. coli, Aeromonas* sp., *A. baumannii*, etc. [34–36]. Presence of different bacteria such as *K. pneumoniae, P. aeruginosa, A. baumannii* along with *E. coli* was found in the maternal samples. However, *mcr-1.1* was found only in *E. coli*, implicating its restricted spread.

In this study, *mcr-*positive isolates belonged to phylogroup A and B1, were highly susceptible and carried few virulence genes. *mcr-1-E. coli* from Southeast Asian countries, also exhibited the prevalence of similar phylogroups. Though primarily restricted to the gut, A and B1 isolates do cause sepsis in debilitated or immunocompromised patients by translocating from gut to blood through the immature/compromised gut barrier [37]. A and B1 *E. coli* being commensals, possess virulence genes necessary for colonization [27], and harbour different ARGs [38] which is also noted among the global *mcr-1* isolates but study isolates exhibited low carriage of ARGs.

*mcr-*positive isolates were diverse and belonged to various STs as also observed in previous studies [39,40]. Occurrence of *mcr-1.1* among novel STs as found in this study, exhibited the emergence of new colistin-resistant clones. This highlighted the fact that the spread of *mcr-1.1* is not through any particular clone or lineage. Southeast Asian *mcr-1* isolates were also diverse irrespective of source of origin. Study isolates exhibited resemblances with isolates from Southeast Asian countries (Bangladesh, Vietnam, Singapore, Laos, etc.).

*mcr-1.1* was detected in IncHI2 and IncX4 replicons, though several other replicons were present in the *mcr-*positive study isolates. Plasmids of this study showed similarities with plasmids from Egypt and Saudi Arabia (IncHI2), and China, Laos, and Vietnam (IncX4), highlighting proficiency of these plasmids for the spread of *mcr* gene. Analysis of Southeast Asian *mcr-1* isolates also exhibited the prevalence of these two plasmids harbouring *mcr-1* gene. IncI2, IncHI2, IncP, and IncX4 are the predominant carriers of *mcr-1* worldwide [27,39,40]. The study isolates shared plasmid backbone similar to replicon type IncHI2. Some differences were noted due to the inversion/deletion of certain sequences. Association of IncHI2-pST4 with *mcr-1.1* in the study isolates, corroborated with others [41,42], implying IncHI2-pST4 to be a dominant plasmid lineage contributing to the horizontal transfer of *mcr-1.1*.

IncHI2 and IncX4 plasmids were conjugative and associated with various MGEs such as IS*Apl1*, Tn*6330* (IncHI2), and IS*26* (IncX4). IS*26* being a hotspot for plasmid fusion has intensified the spread of *mcr-1*-harbouring IncX4 plasmids in the absence of IS*Apl1* or any transposon [43]. IS*Apl1*-*mcr-1.1*-*pap2*-IS*Apl1* is the known genetic background of the gene reported so far [44] but a different genetic environment: IS*Apl1*-*mcr-1.1*-IS*Apl1*-*pap2* has been detected within the study isolates which has not been reported previously.

Clonal spread of *mcr-1.1-E. coli* (ST2705) was observed in three mothers who were admitted in hospital during the same time highlighting the possibility of an inter-mother transmission of *mcr-1.1*-harbouring *E. coli*. Further, similar *mcr-1.1*-plasmids were isolated from five maternal samples in diverse *E. coli.* Each isolate belonged to different STs, but their plasmid sequences revealed significant similarities. Prevalence of different clones with identical plasmids in several mothers indicate that the spread of *mcr-1.1* is plasmid-mediated. However, this study could not definitively underline the fact whether the presence of similar plasmid within different mothers was due to horizontal transmission through bacteria in the hospital environment or independent acquisition of similar plasmids from other sources beyond the hospital environment. In contrast, the transmission of the plasmid in the gut is more evident in cases where similar *mcr-1.1* plasmid has been isolated from different STs in individual mother. Other studies documented the spread of *mcr-1* via plasmids occurring between diarrhoeal patients and from animals to human [39,40,45]. We hypothesize that when more than one *mcr*-positive *E. coli* of distinct STs with similar plasmids were isolated from an individual mother (intra-mother transmission), transfer of *mcr-1.1* via plasmids was more probable than an independent acquisition of two separate distinct *mcr*-possessing isolates.

Presence of *mcr-1.1* in healthy pregnant mothers with no exposure to antibiotics might be due to the presence and persistence of *mcr-1* gene in the food chain. In a recent study, *mcr-1* has been found to enhance the commensal lifestyle of *E. coli*, which led to the maintenance of *mcr-1*-positive *E. coli* within the gut even in the absence of antibiotic pressure [46]. Similarities of *mcr-1-*harbouring maternal study isolates with isolates from chicken (food animal) indicated the presence and circulation of *mcr*-positive isolates within the food chain.

In conclusion, studies highlighting the prevalence of *mcr-1.1* in pregnant mothers are rare with no studies explaining within-host diversity of *mcr-1.1*-harbouring *E. coli*. Presence of *mcr*-positive isolates with highly similar plasmids in the gut of healthy mothers (individual mother or among different mothers) indicated the involvement of plasmids. The only limitation of this study lies in the fact that transmission of *mcr-1.1* from mothers to their respective neonate could not be studied as their babies were healthy and hence not included in the study. The presence of *mcr-1.1* in susceptible *E. coli* of healthy individuals is worrisome since they remain undetected and may serve as a focal point for the spread of colistin resistance in community and in newborns from their colonized mothers.

## Supplementary Material

BasuS_Supplementary_file_230227086R1

## Data Availability

Whole genome sequences of isolates from this study have been submitted to NCBI database under BioProject number: PRJNA808864.
