# Supplementary material for: Carriage and within-host diversity of mcr-1.1-harbouring Escherichia coli from pregnant mothers: inter- and intra-mother transmission dynamics of mcr-1.1
Source: Emerg Microbes Infect. 2023 Dec 17;12(2):2278899. doi: 10.1080/22221751.2023.2278899 (PMC10773534; doi:10.1080/22221751.2023.2278899)
Supplement: BasuS_Supplementary_file_230227086R1 [file TEMI_A_2278899_SM6695.docx]

**Supplementary methods:**

**Processing of rectal swabs/ blood cultures, and detection of transmissible colistin-resistant genes, *mcr*:**

Rectal swabs were plated on chrome agar (BD BBL, MD, USA) supplemented vancomycin (10 mg/L) (MP Biomedicals, California, USA) and incubated at 37ºC for 18-24 hours. Cultures from primary inoculum of each plates were screened for *mcr* genes by polymerase chain reaction (PCR) as described previously [1]. Likewise, blood samples obtained from neonates with suspected sepsis were cultured on blood agar plates (BD BBL, MD, USA) and screened for *mcr* genes by PCR. Samples positive for *mcr* genes were further enriched in Enterobacteriaceae Enrichment (EE) Mossel broth (18-24 hours at 37ºC) (BD BBL, MD, USA) and plated onto chrome agar supplemented with/ without colistin (2 mg/L) (MP Biomedicals, California, USA). Enrichment not only prevented loss of any culturable organism from the specimens, but also helped to retrieve maximum number of organism from both types of plates. This further facilitated assessment of all culturable isolates within the specimen of same patient (mother or neonate) (intra-patient) or among different patients (inter-patient). Different colored colonies were picked from plates with/ without colistin, and again screened for *mcr* genes. Amplified PCR products were processed for Sanger sequencing with primers as described by Ye *et al.* [2]. All possible PCR-positive colonies were glycerol stocked for further use. Any *mcr*-negative colonies with similar color as the *mcr*-positive ones were collected from chrome agar plates (without colistin) and glycerol stocked to compare with *mcr*-positive isolates.

**Whole genome sequencing (WGS) of *mcr-*positive/ *mcr-*negative isolates:**

Distinct isolated colonies of same color with/without the *mcr* gene were cultured onto chromogenic UTI agar (Sigma Aldrich, St. Louis, MO, USA) supplemented with colistin (2 mg/L). DNA extraction and WGS was performed in Cardiff University and genomic DNA (gDNA) was extracted using the QIAamp DNA mini kit using the QIAcube (Qiagen, Germany) with an additional RNAse step. gDNA was quantified using the dsDNA BR assay kit on a Qubit fluorometer 3.0. For short-read sequencing, genomic libraries were prepared using Nextera XT V2 (Illumina, USA), with bead-based normalization. Paired-end WGS was performed on an Illumina MiSeq. Selected isolates (based on gDNA quantity) were subject to additional long-read sequencing utilizing the same gDNA extract which was concentrated and purified at a 1:1 ratio using SPRI beads (Mag-Bind TotalPure, Omega). Genomic libraries were prepared using the Rapid Barcoding Kit (SQK-RBK004; ONT), sequenced on a FLO-MIN106 R9.4 flow cell using a MinION (Oxford Nanopore Technology, UK) and base-called locally with Guppy within MinKnow. Genome assembly and annotation analysis of short-read data has been described fully elsewhere [3]. Briefly, paired end fastq reads were trimmed with the following parameter -phred33 -q 25 --nextera -e 0.2 and reads were assembled using shovill (v0.9.0; --R1, --R2 otherwise default parameters). Genomes either 10% greater than or less than expected genome size for *E. coli* were excluded. Similarly, genomes with >500 contigs (short read only assemblies) were excluded and repeated where possible. *In silico* multilocus sequence typing (MLST) was determined using mlst (v2.17.6) (<https://github.com/tseemann/mlst>). ABRicate (v0.9.7) (<https://github.com/tseemann/abricate>) was used to screen contigs for ARGs using the Resfinder database with a >98% coverage and nucleotide identity applied [4]. For isolates with available long-read data, Unicycler (v0.4.7; -1, -2, -l --mode [normal] parameters applied) was used to generate a hybrid consensus assembly with the corresponding short-reads generated from Illumina. Isolates with novel ST allele profiles were submitted for evaluation using Enterobase (<https://enterobase.warwick.ac.uk/species/ecoli/allele_st_search>).

References:

1. Rebelo AR, Bortolaia V, Kjeldgaard JS, *et al*. Multiplex PCR for detection of plasmid-mediated colistin resistance determinants, *mcr-1, mcr-2, mcr-3, mcr-4* and *mcr-5* for surveillance purposes. Euro Surveill. 2018; 23:17-00672. doi: 10.2807/1560-7917.ES.2018.23.6.17-00672. Erratum in: Euro Surveill. 2018; 23.
2. Ye H, Li Y, Li Z, *et al*. Diversified *mcr-1*-Harbouring Plasmid Reservoirs Confer Resistance to Colistin in Human Gut Microbiota. mBio. 2016; 7:e00177. doi: 10.1128/mBio.00177-16.
3. Sands K, Carvalho MJ, Portal E, *et al*. Characterization of antimicrobial-resistant Gram-negative bacteria that cause neonatal sepsis in seven low- and middle-income countries. Nat Microbiol. 2021; 6:512-523. doi: 10.1038/s41564-021-00870-7.
4. Bortolaia V, Kaas RS, Ruppe E, *et al*. ResFinder 4.0 for predictions of phenotypes from genotypes. J Antimicrob Chemother. 2020; 75:3491-3500. doi: 10.1093/jac/dkaa345.
